# Glossopharyngeal schwannoma causing vocal fold paralysis

**DOI:** 10.1016/S1808-8694(15)30161-0

**Published:** 2015-10-18

**Authors:** Bianca Maria Liquidato, Feres Chaddad Neto

**Affiliations:** a PhD in Otorhinolaryngology at F.C.M. Santa Casa de São Paulo, Professor and Instructor in the Department of Morphology at F.C.M. Santa Casa de São Paulo; b MSc in Neurology at UNICAMP, Assistant Neurosurgeon in the Neurosurgery Program of the Department of Neurology at UNICAMP. F.C.M. da Santa Casa de São Paulo

**Keywords:** glossopharyngeal nerve, schwannoma

## INTRODUCTION

Vocal fold paralysis may occur for a wide range of reasons, from cardiac chamber enlargement, intoxication by chemical products, and mediastinal, neck, lung and intracranial tumors.

Given the vast array of possibilities and the various points where the vagus and recurrent laryngeal nerves (the latter is a branch of the first and innervates most laryngeal intrinsic muscles) may be injured, often times complementary tests are required to reach a diagnosis.

The glossopharyngeal nerve is both a sensitive and motor entity that emerges from the posterior lateral sulcus of the bulb and exits the skull through the jugular foramen. Similarly, the vagus nerve and the cranial root of the accessory nerve emerge from the posterior lateral sulcus of the bulb and exit the skull through the jugular foramen. The hypoglossal nerve emerges from the anterior lateral sulcus of the bulb and exits the skull through the hypoglossal canal. Therefore, expansive lesions affecting any of these nerves may involve the adjacent cranial nerves by compression.

Intracranial schwannomas account for 5-10% of all intracranial tumors^1^. Two to four percent of them are jugular foramen schwannomas^2^. Patients usually present two involved cranial nerves^2^, ^3^, and symptoms do not help much in identifying the nerve of origin^4^. Collet-Sicard syndrome is a rare manifestation that consists of deficits in the four lower cranial nerves, namely the glossopharyngeal, vagus, accessory, and hypoglossal^5^.

## CASE PRESENTATION

Patient SMNS, 55, female, Caucasian, came to our service complaining of closed throat and dysphonia episodes that had been affecting her for the past four months. She reported itchy throat, sensation of pharyngeal globus, and dry cough. She had been seen by an orthopedist to manage pain and reduced strength on the right shoulder.

Physical examination revealed edema of the right supraclavicular region; indirect laryngoscopy showed right para-median vocal fold paralysis. Right vocal fold paralysis consequent to a probable lung apex injury was considered as a diagnostic possibility. The patient was asked to undergo nasal fibroscopy and to have neck, and chest CT scans made. She came back two months later without the ordered tests. Physical examination showed shoulder asymmetry, the right shoulder being higher than the left shoulder, right vocal fold paralysis, and tongue deviated to the right. Diagnostic hypothesis was involvement of X, XI, and XII cranial nerves due to brain tumor. Emergency head MRI scans were ordered.

**Figure 1:**
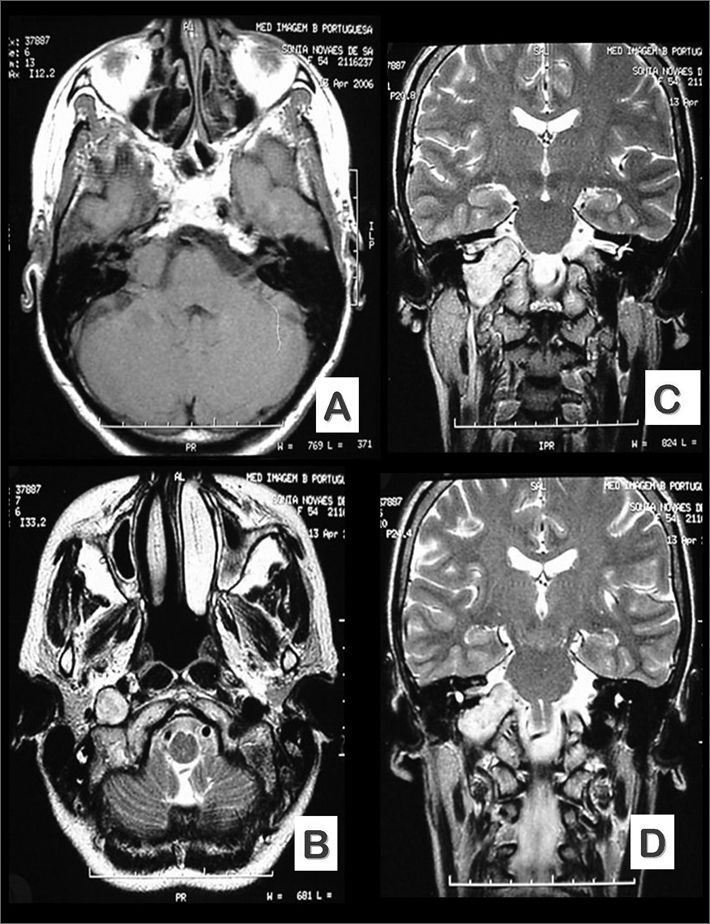
Preoperative MRI images (A and B - axial views, and C and D - coronal views) showing lesion location and size.

The patient”s MRI scans (Fig. 1) showed a tumor in the transition between the bulb and the pons compressing the IX, X, XI, and XII cranial nerves, possibly a schwannoma. She was referred to the neurosurgery and had her jugular foramen operated via the transmastoid approach moving into the skull base. The tumor emerged from the glossopharyngeal nerve was completely resected. Pathology tests revealed the tumor was a schwannoma.

The patient improved from her symptoms with speech therapy, but still 10 months into follow-up she sustains deficits in the affected cranial nerves.

## DISCUSSION

The patient initially reported mild dysphagia, marked dysphonia, and reduced shoulder strength. These symptoms can be related to involvement of at least two cranial nerves, as seen in other reports of jugular foramen schwannoma^2^, ^3^, ^4^. It took the patient a long time to get her tests done and her status evolved to hypoglossal nerve involvement, characterizing a case of Collet-Sicard syndrome^5^. MRI images indicated the presence of a schwannoma, as seen in the literature, but confirmation on the nerve of origin could only be realized intraoperatively^1^, ^4^.

In our case, symptoms connected to glossopharyngeal nerve involvement were quite discrete, and this led the patient to look for our service only when the vagus nerve was compressed and she had dysphonia, a symptom with relevant clinical repercussion. Thus, as described by Di Lazzaro et al.^3^, dysphonia may be one of the main symptoms to look for in jugular foramen schwannomas.

## CONCLUSION

Jugular foramen tumors must be considered in the etiologic diagnosis of vocal fold paralysis, particularly in cases where signs or symptoms associated with the involvement of another cranial nerve are present.
